# Antiproliferative Evaluation of Novel 4-Imidazolidinone Derivatives as Anticancer Agent Which Triggers ROS-Dependent Apoptosis in Colorectal Cancer Cell

**DOI:** 10.3390/molecules27248844

**Published:** 2022-12-13

**Authors:** Jiuhong Huang, Juanli Wang, Guiting Song, Chunsheng Hu, Zhigang Xu, Zhongzhu Chen, Chuan Xu, Donglin Yang

**Affiliations:** 1 College of Pharmacy, National & Local Joint Engineering Research Center of Targeted and Innovative Therapeutics, IATTI, Chongqing University of Arts and Sciences, Chongqing 402160, China; 2 College of Pharmaceutical Sciences, Southwest University, Chongqing 400716, China; 3 Chongqing Academy of Chinese Materia Medica, Chongqing 400065, China; 4 Department of Oncology, Sichuan Cancer Hospital and Institute, Sichuan Cancer Center, School of Medicine, University of Electronic Science and Technology of China, Chengdu 610041, China

**Keywords:** 4-imidazolidinone, colorectal cancer, apoptosis, ROS, JNK

## Abstract

Colorectal cancer (CRC) is one of the most common causes of cancer-related death worldwide, and more therapies are needed to treat CRC. To discover novel CRC chemotherapeutic molecules, we used a series of previously synthesized novel imidazolidin-4-one derivatives to study their anticancer role in several cancer cell lines. Among these compounds, compound **9r** exhibited the best anticancer activity in CRC cell lines HCT116 and SW620. We further investigated the anticancer molecular mechanism of compound **9r**. We found that compound **9r** induced mitochondrial pathway apoptosis in HCT116 and SW620 cells by inducing reactive oxygen species (ROS) production. Moreover, the elevated ROS generation activated the c-Jun N-terminal kinase (JNK) pathway, which further accelerated apoptosis. N-acetylcysteine (NAC), an antioxidant reagent, suppressed compound **9r**-induced ROS production, JNK pathway activation, and apoptosis. Collectively, this research synthesized a series of imidazolidin-4-one derivatives, evaluated their anticancer activity, and explored the molecular mechanism of compound **9r**-induced apoptosis in CRC cells. The present results suggest that compound **9r** has a potential therapeutic role in CRC. Hence, it deserves further exploration as a lead compound for CRC treatment.

## 1. Introduction

Colorectal cancer (CRC) is still one of the most common causes of cancer-related death in China and worldwide [1,2]. Fluorouracil (5-FU) has been pivotal in CRC chemotherapy [3,4]. Recently, biologic agents were introduced for the management of CRC with the aim of signaling cascades involved in tumor growth, metastatic spread, and apoptosis [5]. However, novel chemotherapeutic compounds with biological activity are still needed for CRC treatment.

Apoptosis is programmed cell death in multicellular organisms, which is involved in many cellular mechanisms, such as homeostasis and the elimination of harmful cells. Aberrant apoptosis is fundamental for carcinogenesis, tumor progression, and the development of anticancer drug resistance. It is considered to be a primary target for drug discovery and development, especially for cancer therapies. In the intrinsic apoptotic pathway, proapoptotic protein Bcl-2-associated X (Bax) accumulates at the mitochondrial outer membrane, which causes translocation to the mitochondrial membrane, resulting in mitochondrial outer membrane permeabilization (MOMP) [6]. MOMP leads to the release of cytochrome c, and then triggers the caspase cascade, which results in apoptosis. Anti-apoptotic protein Bcl-2/Bcl-xL mediates the translocation of Bax to cytosol. Consequently, the ratio of endogenous Bcl-2 to Bax determines whether apoptosis occurs or not [7].

The research spectrum of our lab includes synthesizing various of compounds and evaluating their anticancer activity. Through this approach, we have identified some compounds that exhibit prominent anticancer activity [8,9]. Imidazolidin-4-one compounds exist in many natural products, pharmaceuticals, and privileged scaffolds with remarkable anticancer biological activities [10,11,12,13]. We want to synthesis more imidazolidin-4-one compounds with the aim of finding novel compounds with good anticancer activities [14].

The aim of the present study was to evaluate the anticancer activity of our synthesized imidazolidin-4-one compounds and find the most active compound. We found that compound **9r** exhibited prominent anticancer activity in human colorectal cancer cells. We also explored the underlying mechanism of the anticancer role of compound **9r**. The results suggest that compound **9r** is a promising anticancer agent in colorectal cancer, and it is worth further exploring as a lead compound to treat colorectal cancer.

## 2. Results

### 2.1. Anticancer Activity Evaluation of Compounds ***9***

**Scheme 1:**
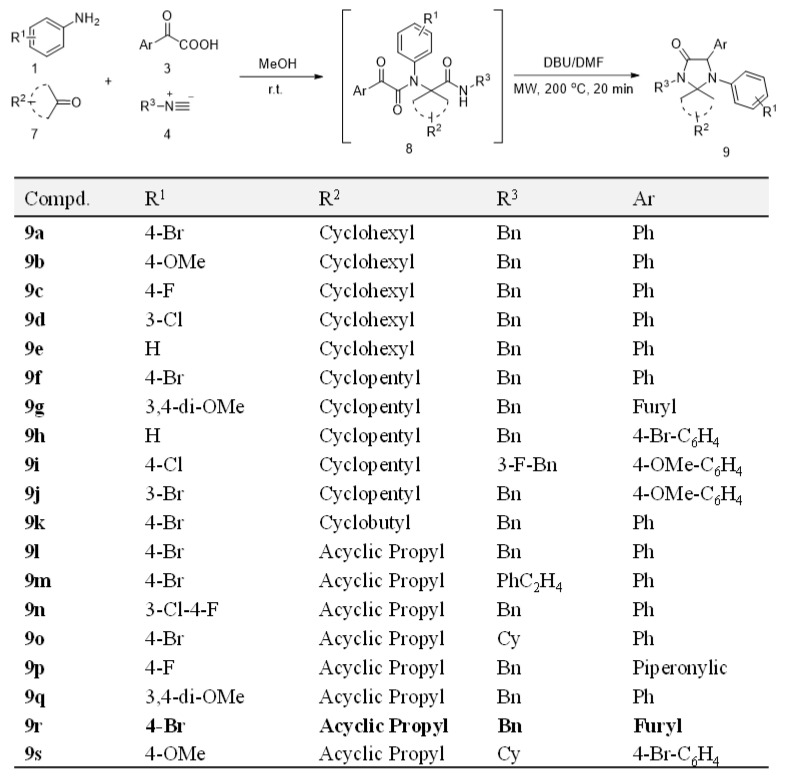
Protocol for the synthesis of compounds **9**.

**Figure 1:**
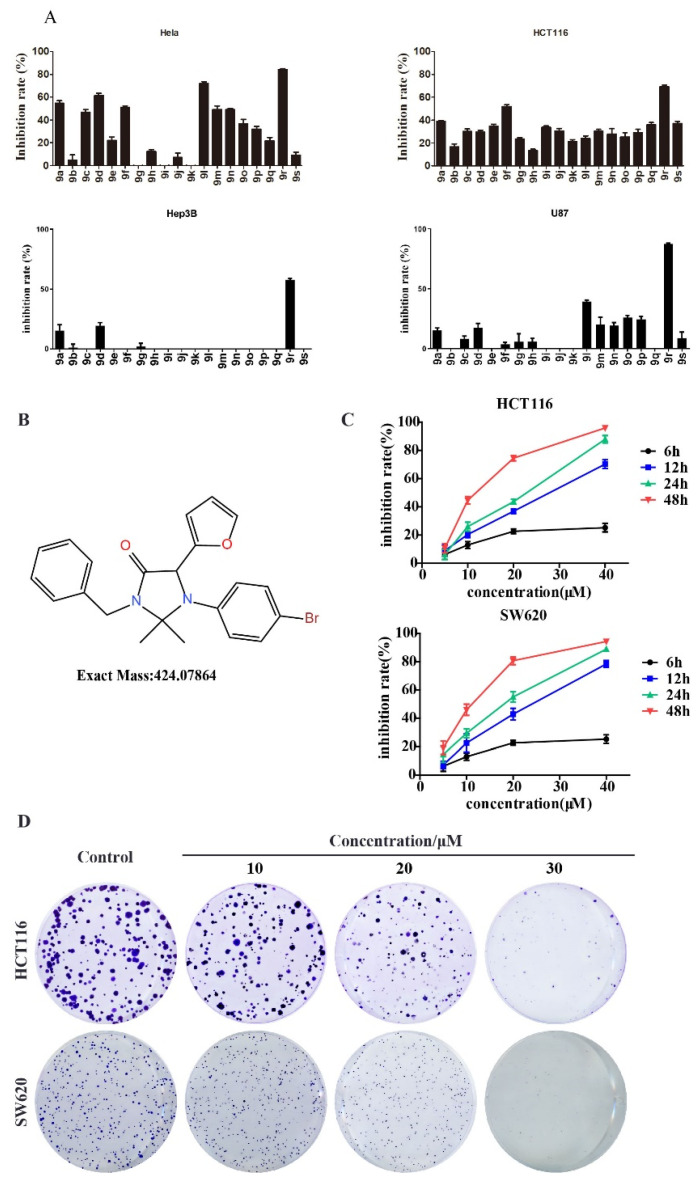
Compounds **9** inhibit tumor cell proliferation and viability. (**A**). Compounds **9** inhibits cancer cell proliferation. Hela, HCT116, Hep3B, and U87 cells were treated with compounds **9** at the concentration of 10 μM for 48 h, cell growth inhibition rate compared with DMSO treatment was measured with MTT assay. Data are represented as mean ± SD (n = 5), the figure only shows the inhibition rate between 0 and 100. (**B**). The chemical structure of compound **9r**. (**C**). HCT116 and SW620 cell were treated with indicated concentrations of compound **9r** for 6, 12, 24 and 48 h; cell growth inhibition rate was measured with MTT assay. Data are demonstrated as the mean ± SD (n = 3). (**D**). Colony formation assay was performed to assess cell growth ability in vitro after treatment with the indicated concentrations of compound **9r**.

In our previous study, we synthesized a small library of 4-imidazolidinones (Scheme 1) [14]. To evaluate the anticancer activity of compounds **9**, we first examined the tumor cell growth inhibition activity in human cancer cell lines. Results from 3-(4,5-dimethylthiazol-2-yl)-2,5-diphenyltetrazolium bromide (MTT) assay showed that compounds **9** inhibited tumor cell growth in cervical adenocarcinoma cells (HeLa), colorectal carcinoma cells (HCT116) and glioblastoma cells (U87). Only a few compounds exhibited anticancer activity in hepatoma cells (Hep3B) (Figure 1A). Some structure–activity relationship (SAR) aspects were investigated in terms of their anticancer activity based on the nature of N-substitution on the 1-imidazolyl ring and the nature of substitution (aliphatic/aromatic) on the 2,5-imidazolyl ring (Scheme 1 and Table S1). The halogenated aromatic substitution on the 1-imidazolyl ring slightly increases anticancer activity ((**9a**, **9c**, **9d**) > **9e**), while the methoxy-conjugated benzene substitution reduced the anticancer activity (**9b** < **9e**). Among the aliphatic substitution on the 2-imidazolyl ring, the influence of substituent on anticancer activity was observed as acyclic propyl > cyclobutyl (**9l** > **9k**). With aromatic substitution on the 5-imidazolyl ring, furyl-substitution significantly enhanced the anticancer activity (**9r** > **9l**).

Among all the compounds, compound **9r** showed the most promising anticancer activity. Therefore, it was selected for further biological studies. Since all compounds **9** had better anticancer activity in HCT116 cells than in other cancer cells (Figure 1A), we selected two colorectal cancer cell lines, HCT116 and SW620, for the following study. The structure of compound **9r** is shown in Figure 1B.

We tested the anticancer activity of compound **9r** in two colorectal cancer cell lines, HCT116 and SW620. The results revealed that the cell growth inhibition rate was elevated as the concentration of compound **9r** increased, and the inhibition rate increased with a fixed concentration of compound **9r** as time passed (Figure 1C). To confirm that compound **9r** was not degraded by esterases, amidases, or other enzymes in the cell culture medium [15], we checked the presence of compound **9r** in cell culture medium with high performance liquid chromatography (HPLC). The results showed that compound **9r** was stable in cell culture medium after 48-hour incubation (Figure S1). Additionally, compound **9r** had low cytotoxicity in normal human colon cells (FHC) (Figure S2). These results indicate that compound **9r** suppresses CRC cell growth in a time- and dosage-dependent manner. The half-maximal inhibitory concentration (IC_50_) of compound **9r** equals 9.44 μM and 10.95 μM in HCT116 and SW620, respectively. The results of the colony formation assay show that compound **9r** suppresses tumor formation in HCT116 and SW620 (Figure 1D). All these results indicate that compound **9r** has good anticancer activity in CRC cells.

### 2.2. Compound ***9r*** Induces Apoptosis

**Figure 2:**
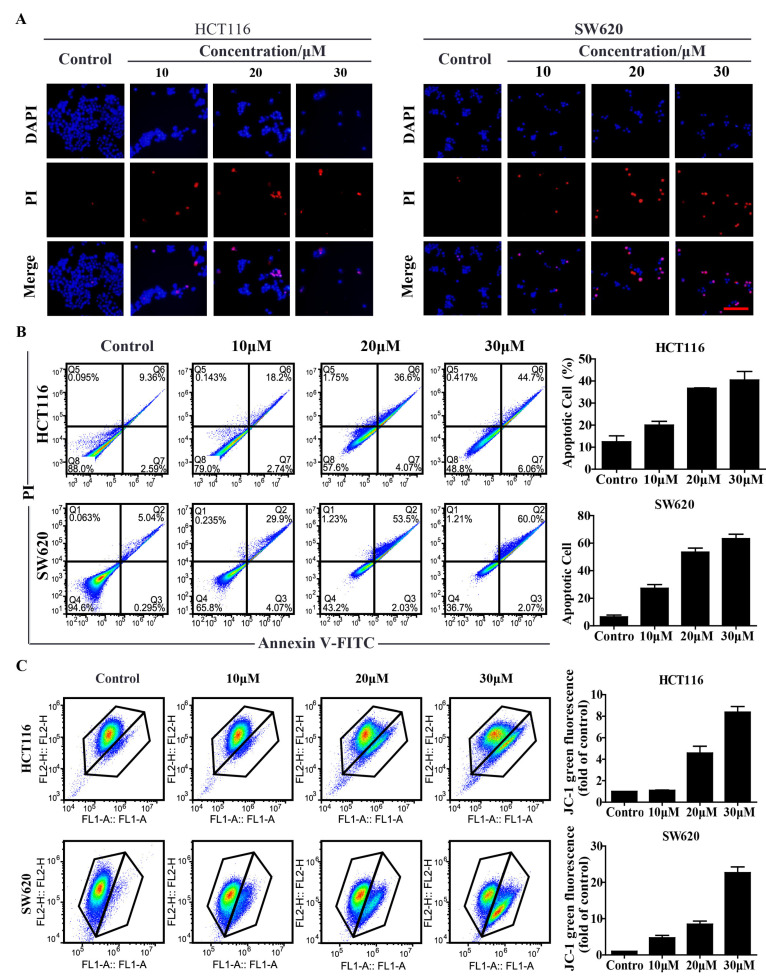
Compound **9r** induces apoptosis in colorectal cancer cells. (**A**). Cell death induced by compound **9r** was observed with a fluorescence microscope after DAPI and PI staining. HCT116 and SW620 cells were treated with indicated concentrations of compound **9r** for 24 h. Cells exhibiting red fluorescence indicated apoptosis or necrosis, the cell nucleus was stained with DAPI. Scale bar represents 100 μm. (**B**). Apoptosis was measured and analyzed via flow cytometry using Annexin V-FITC/PI staining. Cells were treated with the indicated concentration of compound **9r** for 48 h. Bar figure represents the apoptotic rate. Data are represented as mean ± SD (n = 3). (**C**). The mitochondrial membrane potential was measured by flow cytometry using JC-1 staining. Cells were treated with the indicated concentrations of compound **9r** for 24 h. Results indicating the intensity of green fluorescence are shown as fold changes from control from three independent experiments. Data are represented as mean ± SD (n = 3). DAPI: 4′, 6-diamidino-2-phenylindole.

Previous results show that compound **9r** kills all cells at high concentration (Figure 1D). Therefore, we want to know whether compound **9r** induces cell death in colorectal cancer cells. The propidium iodide (PI) staining assay indicated that as the concentration of compound **9r** increased, the cell number decreased and PI positive cells increased (Figure 2A). The results indicate that compound **9r** induces cell death in the cultured colorectal cancer cells of HCT116 and SW620. 

Programed cell death, called apoptosis, is the main reason for chemical reagent-induced cell death in cancer cells. We want to explore whether compound **9r** triggered apoptosis in colorectal cancer cells. Fluorescence-activated cell sorting (FACS) was applied to the colorectal cancer cells of HCT116 and SW620 after compound **9r** treatment. The results revealed that apoptotic cells increased as the concentration of compound **9r** increased in HCT116 and SW620 cells (Figure 2B). These results show that compound **9r** induces apoptosis in colorectal cancer cells.

### 2.3. Compound ***9r*** Triggers Mitochondrial Apoptosis

**Figure 3:**
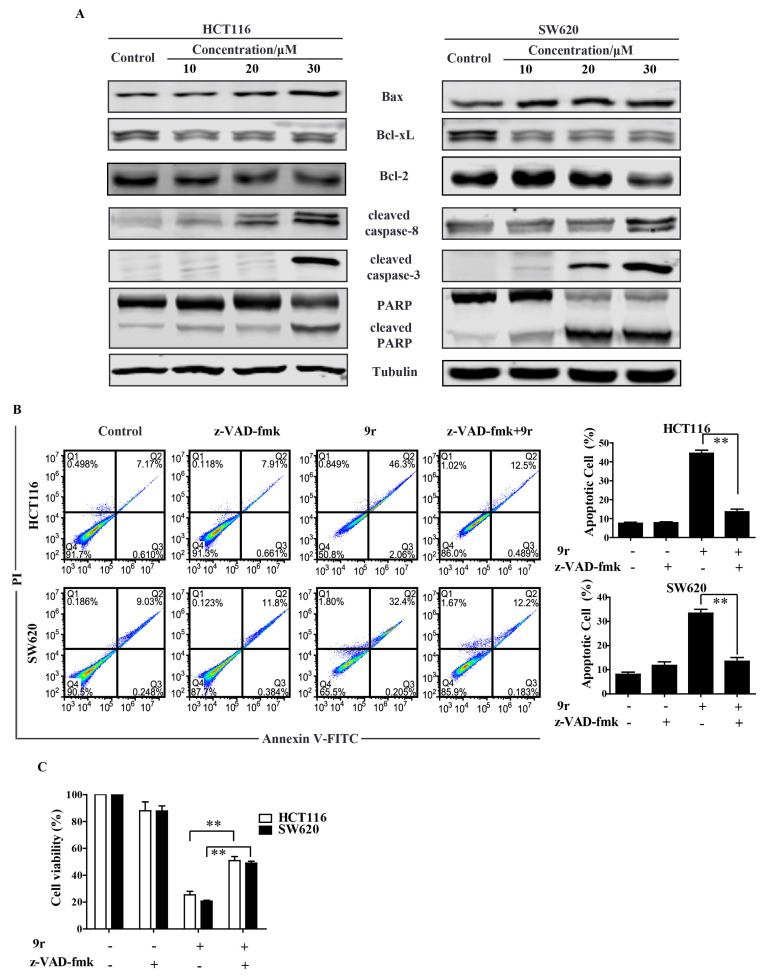
Compound **9r** induces apoptosis through caspase-dependent pathways. (**A**). The expression of apoptotic proteins (Bax, Bcl-xL, Bcl-2, cleaved caspase-8, cleaved caspase-3, PARP and cleaved PARP) was measured using Western blotting, α-Tubulin was used as loading control. Cells were treated with the indicated concentrations of compound **9r** for 48 h. (**B**). Apoptosis was measured by flow cytometry using Annexin V-FITC/PI staining. General caspases inhibitor z-VAD-fmk pretreatment reserved compound **9r**-induced apoptosis in HCT116 and SW620 cells. Cells were pretreated with 50 μM z-VAD-fmk for 2 h before being exposed to 30 μM compound **9r** for 48 h. Bar figure represents the apoptotic rate of indicated compound treatment. Data are represented as mean ± SD (n = 3). Significance was tested by Student’s *t*-test (** *p* < 0.01). (**C**). Cell viability of compound **9r**-treated HCT116 and SW620 cells. Cells were preincubated with 50 μM z-VAD-fmk for 2 h before treatment with 30 μM compound **9r** for 24 h, and the cell viability was measured by MTT assay. Data are demonstrated as the mean ± SD (n = 3). Significance was tested by Student’s *t*-test (** *p* < 0.01).

To explore which pathway was activated to trigger apoptosis by compound **9r**, we examined the mitochondrial membrane potential (ΔΨm) of compound **9r**-treated HCT116 and SW620 cells. Compound **9r** induced the loss of mitochondrial membrane potential (Figure 2C). In accordance with the FACS results, the compound **9r** treatment also increased the cleavage of caspase-8, caspase-3 and poly ADP-ribose polymerase (PARP) (Figure 3A). Compound **9r** also upregulated the expression of proapoptotic protein Bax and down regulated the expression of antiapoptotic proteins Bcl-xL and Bcl-2 (Figure 3A). The ratio of Bax to Bcl-2 was upregulated by the **9r** treatment, which resulted in apoptosis. These results suggest that compound **9r** can trigger mitochondrial apoptosis in colorectal cancer cells.

### 2.4. Compound ***9r*** Induces Caspase-Dependent Apoptosis

Compound **9r** induces the loss of mitochondrial membrane potential, which implies that compound **9r** induces intrinsic apoptosis. To validate whether compound **9r**-triggered apoptosis is caspase-pathway-dependent, we treated HCT116 and SW620 cells with general caspase inhibitor z-VAD-fmk before compound **9r** treatment. The FACS results show that compound **9r**-induced apoptosis is dramatically suppressed by the pretreatment of z-VAD-fmk (Figure 3B). The compound **9r**-induced decrease in cell viability was also suppressed by pretreatment with z-VAD-fmk (Figure 3C). These results demonstrate that compound **9r** induces caspase-dependent apoptosis.

### 2.5. Compound ***9r*** Induces ROS Production

**Figure 4:**
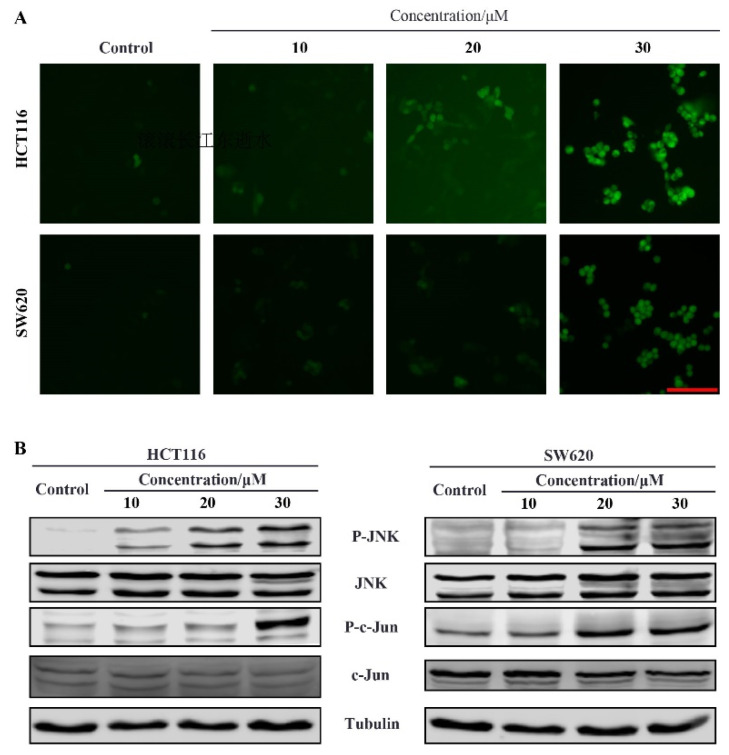
Compound **9r** induces ROS production and activates JNK pathway. (**A**). Fluorescence images of cells stained with DCFH-DA probe. Cells were treated with the indicated concentrations of compound **9r** for 24 h. Scale bar represents 100 μm. (**B**). Expressions of JNK, Phospho-JNK (P-JNK), c-Jun and Phospho-c-Jun (P-c-Jun) were determined by Western blotting after treatment with the indicated concentrations of compound **9r** for 48 h, α-Tubulin was used as a loading control.

Many chemical reagents trigger apoptosis by inducing reactive oxygen species (ROS) production. We want to know whether compound **9r** promoted ROS generation. A DCFH-DA (2′-7′dichlorofluorescin diacetate) probe was used to detect ROS in compound **9r**-treated cells. The results show that the amount of ROS increases as the concentration of compound **9r** is elevated in HCT116 and SW620 cells (Figure 4A).

### 2.6. Compound ***9r*** Induces Apoptosis through ROS Generation Which Activates JNK Pathway

The accumulation of ROS in cells leads to oxidative stress, which activates the c-Jun N-terminal kinase (JNK) pathway. We want to know whether compound **9r** activated the JNK pathway in colorectal cancer cells. Human colorectal cancer HCT116 and SW620 cells were treated with different concentration of compound **9r** for 24 h and subjected to Western blot assay. The results show that, as the concentration of compound **9r** increased, the phosphorylation of JNK and its substrate c-Jun was elevated (Figure 4B), which means that JNK pathway was activated. These results indicate that compound **9r** induces ROS production, which activates JNK pathway.

**Figure 5:**
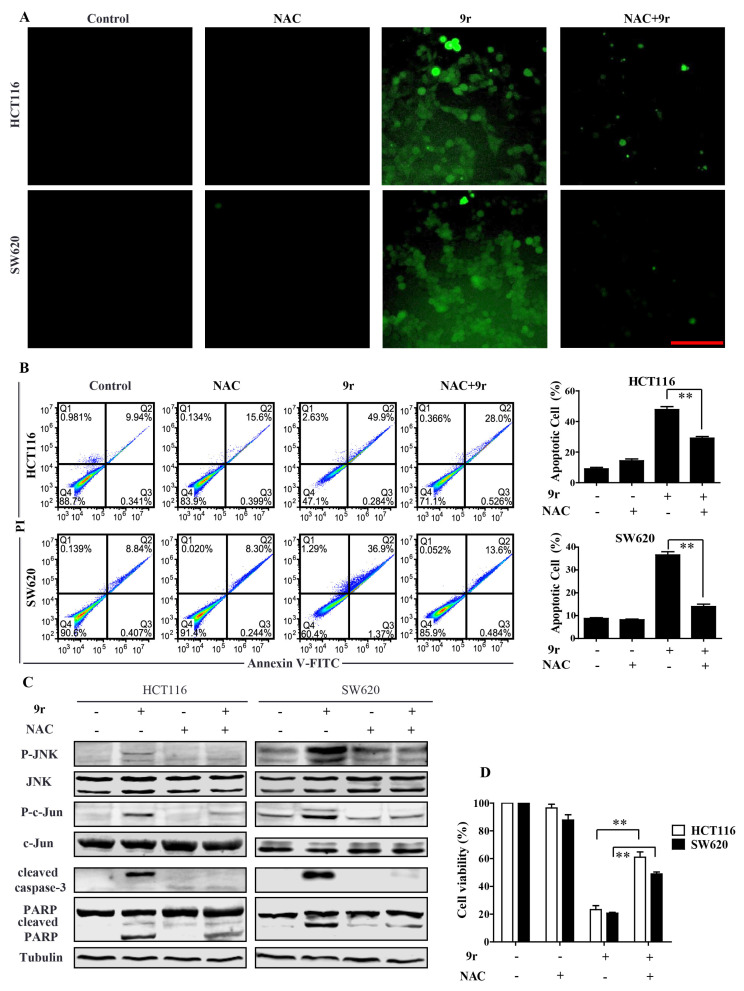
Compound **9r** induces JNK pathway activation and apoptosis through ROS generation. (**A**). Fluorescence images of cells stained with DCFH-DA probe and DAPI. Cells were pretreated with or without 5 mM of NAC for 2 h followed by 30 μM of compound **9r** treatment for additional 24 h. Scale bar represents 100 μm. (**B**). Cell apoptosis was measured by flow cytometry using Annexin V-FITC/PI staining. Cells were pretreated with or without 5 mM of NAC for 2 h, followed by 30 μM of compound **9r** exposure for 24 h. Data represent the apoptosis rate of HCT116 and SW620 cells treated with indicated compound. Bar figures depict the apoptotic rate of indicated compound treatment. Data are represented as mean ± SD (n = 3). Significance was tested by using Student’s *t*-test (** *p* < 0.01). (**C**). Expression of JNK, P-JNK, c-Jun, P-c-Jun, cleaved caspase-3, PARP and cleaved PARP proteins was quantified by Western blotting; α-Tubulin was used as a loading control. HCT116 and SW620 cells were treated with 30 μM of compound **9r** for 24 h with or without 5 mM of NAC pre-treatment. (**D**). Cell viability was measured with MTT assay. Cells were preincubated with 5 mM NAC for 2 h before treatment with 30 μM compound **9r** for 24 h, Data are demonstrated as the mean ± SD (n = 3). Significance was tested by using Student’s *t*-test (** *p* < 0.01).

To find out whether compound **9r**-triggered apoptosis was ROS dependent, we used antioxidant agent *N*-acetylcysteine (NAC) to block ROS generation. The fluorescent images show that compound **9r**-induced ROS production was completely blocked by NAC (Figure 5A). Accordingly, compound **9r**-triggered apoptosis in HCT116 and SW620 cells was suppressed by NAC (Figure 5B). The compound **9r**-activated JNK pathway and caspase pathway were also inhibited by NAC (Figure 5C). Finally, the decrease in compound **9r**-induced cell viability was reversed by NAC in HCT116 and SW620 cells (Figure 5D). All these data imply that compound **9r** induces ROS generation, which activates the JNK pathway and caspase pathway, and finally, the activation of the JNK pathway and caspase pathway results in apoptosis.

**Figure 6:**
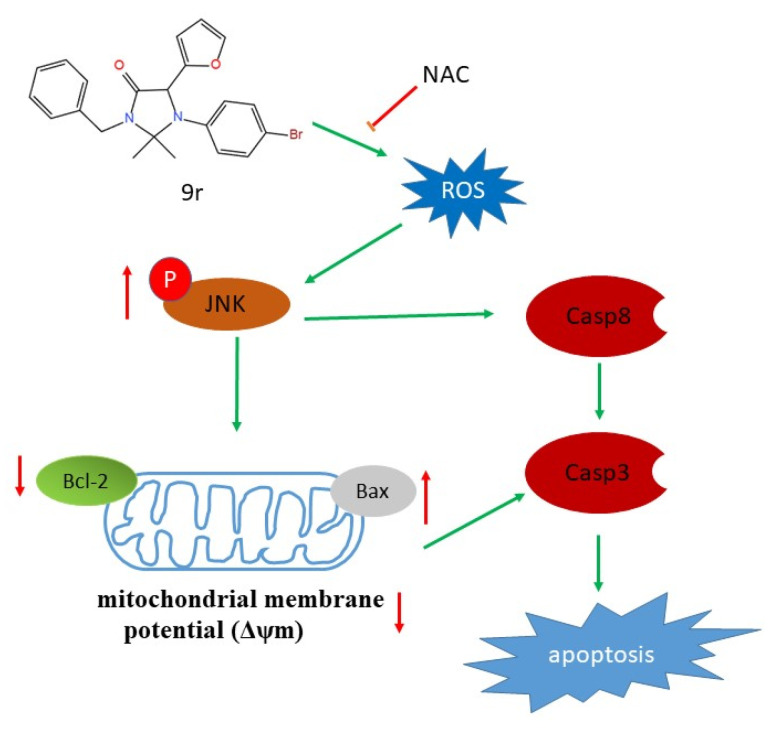
Scheme of this research. Compound **9r** activates JNK pathway by inducing ROS generation. The activated JNK pathway induces the loss of mitochondrial membrane potential, which triggers caspase cascade-mediated apoptosis.

In summary, we synthesized a series of 4-imidazolidinones and evaluated their anticancer activity in various cancer cell lines. From the in-cell anticancer activity screen, we found compound **9r** exhibited the best activity among these compounds. We further investigated the anticancer activity of compound **9r** in colorectal cancer cells. The underlying anticancer mechanism of compound **9r** is that this compound induces ROS accumulation, which activates the JNK pathway and then the caspase pathway, finally resulting in apoptosis (Figure 6). Compound **9r**-triggered ROS generation and caspase-dependent apoptosis were suppressed by NAC.

## 3. Discussion

The compounds with imidazolidinone scaffold have many biological activity [16,17]. A series of imidazolidinone derivatives were reported to be potent phosphodiesterase 4 inhibitors [18]. There has been much research focused on 2-imidazolidinones [19,20,21], but few 4-imidazolidinones have been studied. A previous study reported that 4-imidazolidinone compounds selectively inhibited human neutrophil elastase [22]. A series of 4-imidazolidinone derivatives exhibited modest anticancer cytotoxicity against a variety of cancer cell lines [23]. With the aim of searching for more 4-imidazolidinone compounds with anticancer activity, we synthesized a batch of compounds with a 4-imidazolidinone motif consistent with the previous description [14] and evaluated their anticancer effect in cultured human cancer cells. We also explored the molecular mechanism of anticancer activity of compound **9r,** which harbored the best anticancer effect among these compounds in colorectal cancer cells.

Apoptosis is an important anticancer mechanism induced by many chemotherapies [24]. The intrinsic apoptosis pathway involves Bcl-2 family proteins, including the proapoptotic proteins Bax and Bid, as well as antiapoptotic proteins Bcl-2 and Bcl-xL [25]. Apoptosis is suppressed in CRC as the expression of antiapoptotic Bcl-2 proteins is up regulated [26]. Many small molecules that target Bcl-2 have been identified and used for cancer treatment [27,28,29]. Our study found that compound **9r** increased the expression of Bax, which induced MOMP and then triggered the apoptosis pathway. The expression level of Bcl-2 and Bcl-xL decreased after compound **9r** treatment, which suppressed the antiapoptosis effect of Bcl-2. However, more studies should be carried out to confirm whether compound **9r** targets Bcl-2 to induce apoptosis.

In general, the ROS level is higher in most of cancer cells compared to their normal counterpart cells [30]. Excess ROS generation leads to a loss of mitochondrial membrane potential, resulting in mitochondrial pathway-dependent apoptosis [31]. Many drugs or agents used for the treatment of CRC exert their anticancer effect through ROS-dependent cell death [32]. Our study shows that compound **9r** promotes ROS generation in colorectal cancer cells, which then triggers mitochondrial pathway dependent apoptosis. Furthermore, elevated ROS generation induces oxidative stress that activates the JNK pathway, which is well-known as a stress-activated protein kinase which leads to apoptosis. Taken together, compound **9r** induces ROS accumulation in cells, which leads to MOMP and JNK pathway activation, and finally results in apoptosis. Similar to previously reported agents used to treat CRC, compound **9r** exerts an anticancer role by promoting ROS generation.

In summary, we used a batch of previously synthesized imidazolidin-4-one compounds to explore their anticancer activity. The in vitro experiments show that compound **9r** plays a prominent role in inducing cell death in colorectal cancer HCT116 cells and SW620 cells. The compound **9r** treatment increases ROS generation, which subsequently leads to the upregulation of Bax/Bcl-2 and the activation of the mitochondrial-dependent apoptotic pathway. Moreover, compound **9r** also activates JNK pathway, which induces apoptosis. 

## 4. Materials and Methods

### 4.1. Chemical Synthesis of Compounds **9**

Previously, we reported a post-Ugi/decarboxylative C(*sp*^3^)-N bond formation cascade reaction that proceeds under microwave irradiation to construct 4-imidazolidinones and drug-like spiroimidazolones [14]. Compounds **9** were synthesized as previously reported (Scheme 1) [14].

As part of our continued efforts to develop multicomponent reaction for therapeutic development [33], we reported a novel post-Ugi 4-component (U-4CR) cascade reaction for the synthesis of 4-imidazolones as previously reported [14]. We initially investigated the scope of the reaction to prepare a small library of 4-imidazolidinones. In all cases, initial Ugi products were obtained in good yields following the removal of the reaction solvent without further purification. A variety of different starting materials were successfully employed for the construction of structurally diverse 4-imidazolidinones with good yields, indicating a good functional group tolerance (Scheme 1). 

### 4.2. Cell Culture

The human cervical adenocarcinoma cells of Hela, colorectal carcinoma cells of HCT116 and SW620, hepatoma cells of Hep3B, glioblastoma cells of U87, and fetal colon cells of FHC were obtained from Cobier Biotechnology (Cobier, Nanjing, China). U87 cells, SW620 cells, and Hep3B cells were cultured in high-glucose Dulbecco’s modified eagle medium (DMEM, Hyclone, Logan, UT, USA) supplemented with 10% fetal bovine serum (FBS, Gibco, Australia origin). Hela cells were cultured in Roswell Park Memorial Institute (RPMI) 1640 medium (Hyclone, Logan, UT, USA) with 10% FBS. HCT116 cells were cultured in McCoy’s 5A medium (Hyclone, Logan, UT, USA) supplemented with 10% FBS. FHC cells were cultured in DMEM:F12 medium supplemented with 10% FBS, 0.005 mg/mL insulin and 20 ng/mL human recombinant EGF. Cells were maintained at 37 °C under a humidified atmosphere and 5% CO_2_.

### 4.3. Reagents and Antibodies

The chemical reagents used in this study, 3-[4,5-dimethylthiazol-2-yl]-2,5-diphenyl-tetrazolium bromide (MTT), NAC, DAPI, PI, and DMSO, were purchased from Sigma-Aldrich. The inhibitor z-VAD-fmk was purchased from Beyotime Biotechnology. The primary antibodies, Bax, Bcl-2, Bcl-xL, cleaved caspase-8, caspase-3, cleaved caspase-3, PARP, cleaved PARP, P-JNK, JNK, P-c-Jun, c-Jun, and α-Tubulin were purchased from Cell Signaling Technology.

### 4.4. MTT Assay

Cells were seeded into wells of a 96-well plate at 4 × 10^3^ cells per well in 100 µL of the corresponding medium. After incubation for 24 h, cells were treated with compound **9r** at various concentrations, followed by continuous incubation for 24, 48, or 72 h. A 20 μL MTT solution was directly added to each well, and the incubation was continued for an additional 4 h. Finally, the purple formazan crystals were dissolved in DMSO. The absorbance was measured with a microplate reader (Bio-Tek, Winooski, VT, USA) at a wavelength of 570 nm.

### 4.5. Colony Formation Assay

Cells at a density of 600 cells per well were seeded into a 6-well plate. After an incubation of 24 h, cells were treated with compound **9r** at concentration of 0, 10, 20 and 30 µM for 48 h. Cells were cultured with fresh medium for an additional 14 days. Then, the media was removed, and cells were washed with PBS thrice and fixed with 4% paraformaldehyde for 20 min at room temperature. Cells were washed with PBS thrice and stained with 1% crystal violet for 30 min. After the crystal violet was removed, the plate was rinsed with PBS and allowed to air dry. Cells were imaged with a scanner (Epson Perfection V800 Photo).

### 4.6. DAPI/PI Staining Assay

Cells were evenly dispersed on 12-well plates at a density of 2 × 10^4^ cells per well. After being incubated overnight, the cells were treated with compound **9r** at different concentrations for 24 h. Then, the cells were washed thrice with PBS and fixed with 4% Paraformaldehyde for 20 min at room temperature, followed by staining with DAPI (10 μg/mL) and PI (5 μg/mL) for 30 min. The images of the stained cells were taken using an inverted fluorescence microscope (IX73 Olympus). 

### 4.7. Apoptosis Analysis

Cells were seeded into 6-well plates at 2 × 10^5^ cells per well, incubated overnight, and treated with compound **9r** at various concentrations for 48 h. Cells were harvested with 0.1% trypsin and washed with cold PBS twice, which was followed by resuspension in binding buffer. Afterwards, 5 μL of Annexin V fluorescein isothiocyanate (Annexin V-FITC) and 10 μL of propidium iodide (PI) were added, the mixture was kept in the dark for 15 min at room temperature, and finally, 200 μL of binding buffer was added. The cells were analyzed immediately by using flow cytometry (BD Accuri C6).

### 4.8. Mitochondrial Membrane Potential Assay

Cells were seeded into 6-well plates at a density of 2 × 10^5^ cells per well and incubated overnight. After treatment with different concentrations of compound **9r** for 24 h, the cells were harvested with 0.1% trypsin and washed twice with cold PBS. Cells were then suspended in 1 × JC-1 staining buffer (Beyotime Biotechnology, Shanghai, China) and incubated at 37 °C for 30 min in the dark. The relative fluorescence intensity of each sample was analyzed using flow cytometry with the setting of FL1A at 530 nm and FL2H 585 nm.

### 4.9. Measurement of Intracellular ROS

The level of intracellular ROS was estimated quantitatively using a peroxide-sensitive fluorescent probe, DCFH-DA (2′,7-dichlorofluorescein diacetate), which is oxidized in the presence of peroxides to the highly fluorescent DCF (2′,7-dichlorofluorescein). Cells were seeded into a 12-well plate at a density of 2 × 10^4^ cells per well. After being incubated overnight, cells were treated with compound **9r** at different concentrations for 24 h in the absence or presence of 5 mM of NAC for 2 h at 37 °C. The cells were then treated with 10 μM DCFH-DA for 30 min at 37 °C. The images of the stained cells were immediately taken with an inverted fluorescence microscope.

### 4.10. Western Blotting

Cells were harvested after compound **9r** treatment, and then lysed in a RIPA buffer containing protease and phosphatase inhibitors. Lysates were quantified using the BCA assay kit (Beyotime Biotechnology). A total of 50 µg proteins was separated by SDS-PAGE and transferred onto polyvinyldene difluoride (PVDF, Millipore Corporation, Burlington, MA, USA) membranes afterward. Membranes were blocked with 1 × QuickBlock™ Blocking Buffer (Beyotime Biotechnology) for 1 h, which was followed by incubation with specific primary antibodies at 4 °C overnight and fluorescence-conjugated secondary antibody at room temperature for 1 h. Immunoreactive proteins were visualized using the Odyssey Fluorescence Scanner.

## 5. Conclusions

The present study shows that compound **9r** induces apoptosis in the colorectal carcinoma cells HCT116 and SW620. The underlying mechanism of compound **9r** was found to induce ROS generation, which triggers apoptosis and the activation of the JNK pathway. These results provide a novel insight, revealing that imidazolidin-4-one compounds exert an anticancer effect by inducing ROS-mediated apoptosis. The compound **9r** is worthy of further exploration as a lead compound to treat colorectal cancer.

## Data Availability

Not applicable.

## Supplementary Materials

The following supporting information can be downloaded at: https://www.mdpi.com/article/10.3390/molecules27248844/s1, Figure S1: HPLC chromatograms of standards of compound **9r**. Figure S2: FHC cell viability after compound **9r** treated for 72 h; Table S1: The inhibition rate of compounds **9** in tumor cells.

## Sample Availability

Samples of the compounds **9** are available from the authors.
