# Supplementary material for: Antiproliferative Evaluation of Novel 4-Imidazolidinone Derivatives as Anticancer Agent Which Triggers ROS-Dependent Apoptosis in Colorectal Cancer Cell
Source: Molecules. 2022 Dec 13;27(24):8844. doi: 10.3390/molecules27248844 (PMC9783213; doi:10.3390/molecules27248844)
Supplement: Supplementary file 1 [file molecules-27-08844-s001.zip › molecules-2054957-supplementary.pdf]

Antiproliferative evaluation of novel 4-imidazolidinone derivatives as anticancer agent which triggers ROS-dependent apoptosis in colorectal cancer cell

Jiuhong Huang, Juanli Wang, Guiting Song, Chunsheng Hu, Zhigang Xu, Zhongzhu Chen, Chuan Xu, Donglin Yang

Table S1. The inhibition rate of compounds 9 in tumor cells.

Compound 9					Inhibition rate (%)			
Compound	R[1]	R[2]	R[3]	Ar	HCT116	Hela	Hep3B	U87
**9a**	4-Br	Cyclohexyl	Bn	Ph	38.9±1.9	54.6±5	15.1±11.3	15.4±4.4
**9b**	4-OMe	Cyclohexyl	Bn	Ph	16.7±5.5	4.6±10.8	1±6.4	NA
**9c**	4-F	Cyclohexyl	Bn	Ph	30.1±5.1	46.7±5.1	NA	8.3±5
**9d**	3-Cl	Cyclohexyl	Bn	Ph	29.4±3.8	61.5±4.4	19.4±5.6	17.5±8.2
**9e**	H	Cyclohexyl	Bn	Ph	34.8±3.6	22±7	NA	NA
**9f**	4-Br	Cyclopentyl	Bn	Ph	51.9±3.4	51.2±2.8	NA	3.8±3.2
**9g**	3,4-di-OMe	Cyclopentyl	Bn	Furyl	23.7±2.4	NA	1.8±6	5.6±15.5
**9h**	H	Cyclopentyl	Bn	4-Br-C_6H_4	13.3±2.9	12.5±3.5	NA	5.8±6.3
**9i**	4-Cl	Cyclopentyl	3-F-Bn	4-OMe-C_6H_4	33.6±2.9	NA	NA	NA
**9j**	3-Br	Cyclopentyl	Bn	4-OMe-C_6H_4	30.5±5	7.1±8.9	NA	NA
**9k**	4-Br	Cyclobutyl	Bn	Ph	21.4±3.7	NA	NA	NA
**9l**	4-Br	Acyclic Propyl	Bn	Ph	24.1±4.9	72±3.7	NA	39.3±3
**9m**	4-Br	Acyclic Propyl	PhC_2H_4	Ph	30.4±3.5	49.2±7.1	NA	20.3±13.2
**9n**	3-Cl-4-F	Acyclic Propyl	Bn	Ph	27.7±11.2	49±2.7	NA	19.2±5.8
**9o**	4-Br	Acyclic Propyl	Cy	Ph	25.1±8.9	36.7±8.5	NA	26.1±3.9
**9p**	4-F	Acyclic Propyl	Bn	Piperonylic	29.1±6.5	31.8±6.5	NA	24.5±5.6
**9q**	3,4-di-OMe	Acyclic Propyl	Bn	Ph	35.9±4.6	21.7±7.1	NA	NA
**9r**	4-Br	Acyclic Propyl	Bn	Furyl	69.3±3.1	84.1±2.1	57.7±2.4	87.5±1.5
**9s**	4-OMe	Acyclic Propyl	Cy	4-Br-C_6H_4	37.1±4.4	9.2±6.1	NA	8.9±10.9

The inhibition rate is described as “NA” if the value is below 0.

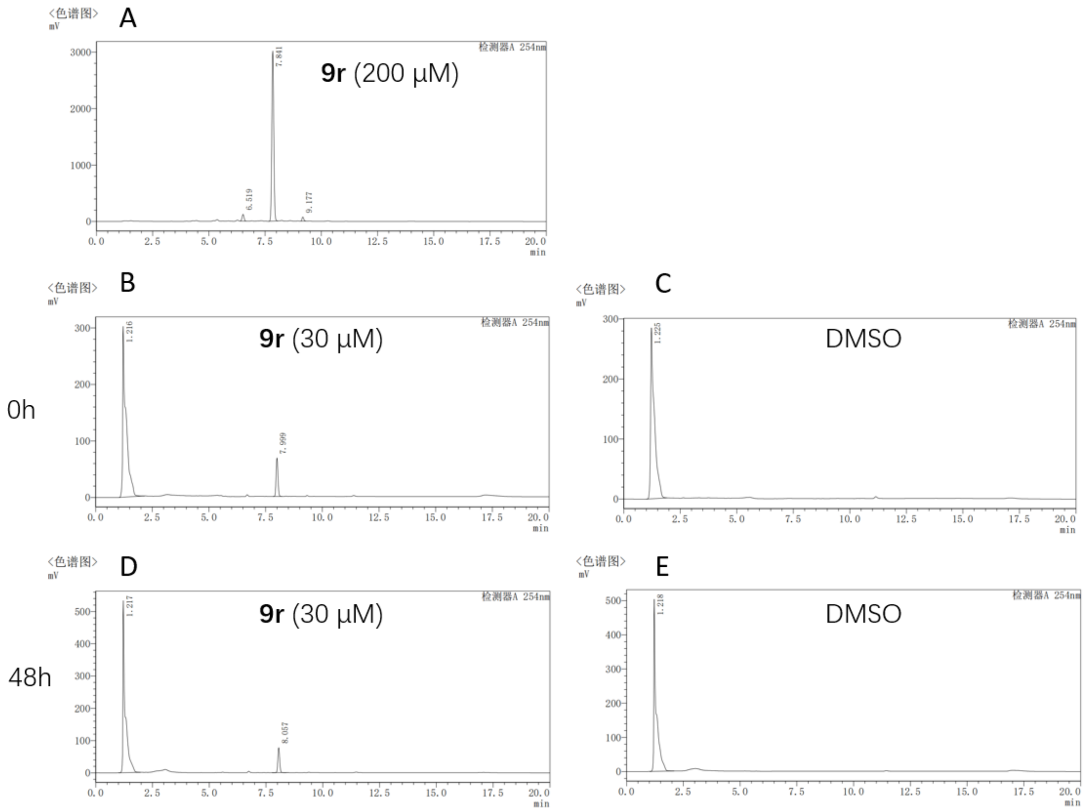

Figure S1. HPLC chromatograms of standards of compound **9r** (200 μM in methanol) (A), 30 μM compound **9r** in medium containing 10% FBS (B), DMSO in medium containing 10% FBS (C), 30 μM compound **9r** in medium containing 10% FBS after 48 hours of incubation (D), DMSO in medium containing 10% FBS after 48 hours of incubation (E). HPLC: High performance liquid chromatography, DMSO: Dimethyl sulfoxide, FBS: Fetal bovine serum.

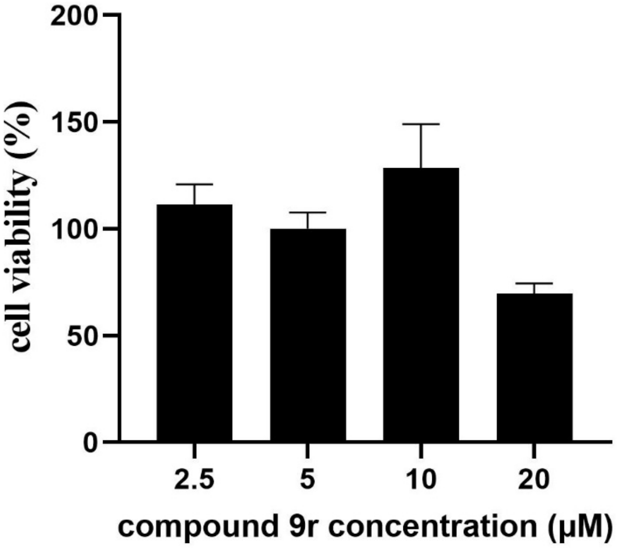

Figure S2. FHC cell viability after compound **9r** treated for 72 hours.
